# Supplementary material for: The potential of novel bacterial isolates from healthy ginseng for the control of ginseng root rot disease (Fusarium oxysporum)
Source: PLoS One. 2022 Nov 10;17(11):e0277191. doi: 10.1371/journal.pone.0277191 (PMC9648753; doi:10.1371/journal.pone.0277191)
Supplement: Attachment — Submitted filename: 2nd Reviwer.docx [file pone.0277191.s002.docx]

The manuscript was re-write well based on suggestion of reviewer, but reviewer think that the authors should be mention the accession number in the abstract and the methods.
