# Supplementary material for: The potential of novel bacterial isolates from healthy ginseng for the control of ginseng root rot disease (Fusarium oxysporum)
Source: PLoS One. 2022 Nov 10;17(11):e0277191. doi: 10.1371/journal.pone.0277191 (PMC9648753; doi:10.1371/journal.pone.0277191)
Supplement: Attachment — Submitted filename: Response to Reviewers.docx [file pone.0277191.s003.docx]

**Response to Editor and Referees**

**Editor：**

**Please review your reference list to ensure that it is complete and correct. If you have cited papers that have been retracted, please include the rationale for doing so in the manuscript text, or remove these references and replace them with relevant current references. Any changes to the reference list should be mentioned in the rebuttal letter that accompanies your revised manuscript. If you need to cite a retracted article, indicate the article’s retracted status in the References list and also include a citation and full reference for the retraction notice.**

**Response:** We sincerely thank your professional comments and careful checks of our study. At your suggestion, to avoid citing papers that have been retracted, we have re-cited the relevant literature, as indicated by the red markings in the references.

**Reviewer #1：**

**In section 3.2 (Gene bank sequence registration number AF077393.1), change it to
Accession number: AF077393**

**Response:** We are grateful for your valuable suggestions. In our resubmitted manuscript, (Gene bank sequence registration number AF077393.1) has been changed to Access number: AF077393 according to your suggestion.

**Change Similarity to Identity in all the text**

**Response:** We sincerely thank your professional comments and careful checks of our study. According to your suggestion, we have corrected the manuscript and marked the revised portions in red.

**Revise the sections on molecular and morphological identification sections numbering is missing**

**Response:** Thanks for your constructive suggestions. We have added the numbering on molecular and morphological identification sections and marked the revised portions in red.

**Reviewer #2:**

**The manuscript was re-write well based on suggestion of reviewer, but reviewer think that the authors should be mention the accession number in the abstract and the methods.**

**Response:** We extremely grateful to your visionary comments. According to your comments, we have added the accession number in the abstract and the methods.
